# Measures of anxiety, depression and stress in the antenatal and perinatal period following a stillbirth or neonatal death: a multicentre cohort study

**DOI:** 10.1186/s12884-021-04289-0

**Published:** 2021-12-10

**Authors:** Suzanne Thomas, Louise Stephens, Tracey A. Mills, Christine Hughes, Alan Kerby, Debbie M. Smith, Alexander E. P. Heazell

**Affiliations:** 1 Saint Mary’s Hospital, Manchester University NHS Foundation Trust, Oxford Road, Manchester, M13 9WL UK; 2 Liverpool School of Tropical Medicine, Pembroke Place, Liverpool, L3 5QA UK; 3 Maternal and Fetal Health Research Centre, Division of Developmental Biology and Medicine, Faculty of Biology, Medicine and Health, University of Manchester, 5th floor (Research), St Mary’s Hospital, Oxford Road, Manchester, M13 9WL UK; 4 Manchester Centre for Health Psychology, School of Health Sciences, Faculty of Biology, Medicine and Health, University of Manchester, Manchester, UK

**Keywords:** Perinatal Death, Stillbirth, Neonatal Death, Subsequent Pregnancy, Pregnancy after loss, Anxiety, Depression, Perceived Stress

## Abstract

**Background:**

The grief associated with the death of a baby is enduring, however most women embark on another pregnancy, many in less than a year following their loss. Symptoms of anxiety and depression are reported to be increased in pregnancies after perinatal death, although effect on maternal stress is less clear. Variation between individual studies may result from differences in gestation at sampling, the questionnaire used and the type of antecedent perinatal death. We aimed to describe quantitative measures of anxiety, depression, stress and quality of life at different timepoints in pregnancies after perinatal death and in the early postnatal period.

**Methods:**

Women recruited from three sites in the North-West of England. Women were asked to participate if a previous pregnancy had ended in a perinatal death. Participants completed validated measures of psychological state (Cambridge Worry Score, Edinburgh Postnatal Depression Score (EPDS), Generalized Anxiety Disorder 7-item score) and health status (EQ-5D-5L™ and EQ5D-Visual Analogue Scale) at three time points, approximately 15 weeks’ and 32 weeks’ gestation and 6 weeks postnatally. A sample of hair was taken at approximately 36 weeks’ gestation for measurement of hair cortisol in a subgroup of women. The hair sample was divided into samples from each trimester and cortisol measured by ELISA.

**Results:**

In total 112 women participated in the study. Measures of anxiety and depressive symptoms decreased from the highest levels at 15 weeks’ gestation to 6-weeks postnatal (for example mean GAD-7: 15 weeks 8.2 ± 5.5, 6 weeks postnatal 4.4 ± 5.0, p<0.001). Hair cortisol levels fell in a similar profile to anxiety and depression symptoms (p<0.05). In contrast, the median EQ-5D index, measuring health status was 0.768 at 15 weeks’ gestation (Interquartile range (IQR) 0.684-0.879), 0.696 at 32 weeks’ (IQR 0.637-0.768) and 0.89 (0.760-1.00) at 6 weeks postnatal. There was a negative relationship between EPDS and perceived health status.

**Conclusions:**

This study demonstrated heightened anxiety and depressive symptoms and elevated cortisol levels in women in pregnancies after a stillbirth or neonatal death which decrease as pregnancy progresses. Further studies are needed to determine optimal care for women to address these negative psychological consequences.

## Background

In the UK, approximately 1 in 250 babies are stillborn and 1 in 600 babies die in the first month of life; thus, 4,100 families are bereaved each year [1]. The death of a baby before or shortly after birth (hereafter referred to as perinatal death) is a profoundly distressing experience for women and their families and is invariably followed by a period of grief [2]. Although grieving, women frequently report planning for another pregnancy [3], and most will embark on another pregnancy, with estimates of 50% of women conceiving within a year and 86% within 18 months [4, 5]. Previous perinatal death is consistently recognised to increase parents’ anxiety, perceived stress, emotional vulnerability and decrease their confidence in outcome of the next pregnancy [6, 7]. Hunter et al. performed a systematic review, including 19 quantitative studies describing anxiety, depression and stress in a meta-analysis. This study found an association between anxiety and depression in a subsequent pregnancy, but there was significant heterogeneity between studies, potentially due to differences in the psychometric measurement tools used, gestation at sampling and the nature of previous perinatal loss being studied. The authors concluded that “more targeted studies that examine the effect of specific perinatal loss experiences may be beneficial in terms of identifying variations in women's need for support during subsequent pregnancies.”

The reported increase in anxiety and stress is a cause for concern because this increases the risk of adverse pregnancy outcomes, notably preterm birth and low birthweight [8]. Furthermore, a longitudinal study reported that the negative psychological impacts of perinatal death persist beyond the next pregnancy despite the birth of a healthy child [9]; previous history of perinatal death has been reported to disrupt maternal attachment and negatively impact on parenting [10]. Therefore, better appreciation of the profile of symptoms of anxiety, depression and psychological stress would enable maternity services to focus on women’s needs in and after pregnancy after perinatal death. Therefore, this study aimed to recruit women in pregnancies after perinatal death to describe quantitative measures of anxiety, depression, stress and quality of life at different time points in their pregnancies and in the early postnatal period.

## Methods

Following ethical approval (Ref 16/NW/0258) the study was conducted between 22/09/2016 and 21/12/2018 at three sites: St Mary’s Hospital and Wythenshawe Hospital, Manchester and Royal Preston Hospital, UK. At the first two sites there was a specialist service (Rainbow Clinic) for care after perinatal death, the model of care follows the international consensus statement for care in pregnancies after stillbirth [11]. At the third site there was a dedicated bereavement midwife to support families following the death of their baby. Pregnant women were eligible for inclusion if they were attending the antenatal service for care in a pregnancy after perinatal death (stillbirth or neonatal death). Women were excluded if they were less than 16 years of age, lacked capacity to consent or who had been diagnosed with pregnancy complications or received treatment for an acute mental health issue in this pregnancy.

### Completion of Validated Questionnaires

After providing written informed consent participants were asked to complete a questionnaire booklet which contained the Cambridge Worry Score (CWS) [12], Edinburgh Postnatal Depression Score (EPDS) [13], Generalised Anxiety and Depression 7-item (GAD-7) score [14]), EuroQoL-5 dimension (EQ-5D-5L™), a standardised instrument for use as a measure of health related quality of life and EQ-5D Visual Analogue Scale [15]. All questionnaires were in English. Demographic characteristics were obtained from participant’s maternity case notes. The completed questionnaires were given to the researcher or returned by mail. Participants were requested to complete the questionnaires again at 32 weeks’ gestation and 6 weeks postpartum. At the latter time point the questionnaire booklet was sent by mail and returned by the participant.

Responses from each timepoint were scored using the appropriate scoring matrix at the completion of the study and entered into a study database. The EQ-5D-5L is measured on five dimensions (mobility, self-care, usual activities, pain/discomfort and anxiety/depression), each with five levels (no problems, slight problems, moderate problems, severe problems and extreme problems). This allows participants to be classified into one of 3,125 health states to which appropriate utility weights can then be attached and the EQ-5D Index can be calculated. The values generated generally range from 0 (no heath) to 1 (full health). The proportion of GAD-7 scores >10 were taken to indicate significant anxiety [14] and EPDS >13 were taken to indicate significant depressive symptoms [16]. A threshold was not applied to the CWS results as one was not identified from the literature.

### Hair Cortisol Measurements

A 0.5 cm diameter of hair at the vertex of the head was cut at the scalp and stored at -20 °C. The hair was trimmed down to 13 cm from the scalp end and washed in high purity isopropanol on a rotator for 3 minutes. After being air-dried at room temperature for 2 days, it was cut into consecutive 3 cm samples representing hair growth from each trimester. 60 mg of hair was minced with a scalpel and ground in a bead beater for 5 minutes (Bullet Blender, Next Advance). Ground hair samples were incubated in 1.5 ml of high purity methanol for 18 hours at room temperature on a rotator. After being centrifuged at 10,000 rpm for 5 minutes 1 mL of the supernatant was transferred to a tube and dried in a vacuum evaporator (MiVac, Genevac). The extract was re-suspended in 250 μl phosphate buffered saline. Samples were assayed in duplicate using the standard protocol from a cortisol ELISA kit (ALPCO) and measured at 450 nm in a spectrophotometer (Omega, BMG Labtech). Duplicate measurements with a coefficient of variation greater than 40% were excluded from the analysis. Sample cortisol measurements were normalised for sample hair weight.

### Statistical analysis

Quantitative data were entered into Microsoft Excel for descriptive statistical analysis. Comparative analysis was undertaken in STATA Version 14 (StataCorp, TX, USA) to determine whether there were differences in questionnaire scores or cortisol levels at different gestations of pregnancy or postnatal. Distribution of data was evaluated by Shapiro-Wilk test. Normally distributed data were analysed by one-way ANOVA and non-normally distributed data were analysed by Kruskal-Wallis test, matched data were analysed by repeated measures ANOVA or Friedman test depending on whether the data were normally distributed. Due to an absence of preliminary data in this population a formal sample size calculation was not performed to inform study size. A p value of <0.05 was taken to indicate statistical significance.

## Results

**Fig. 1:**
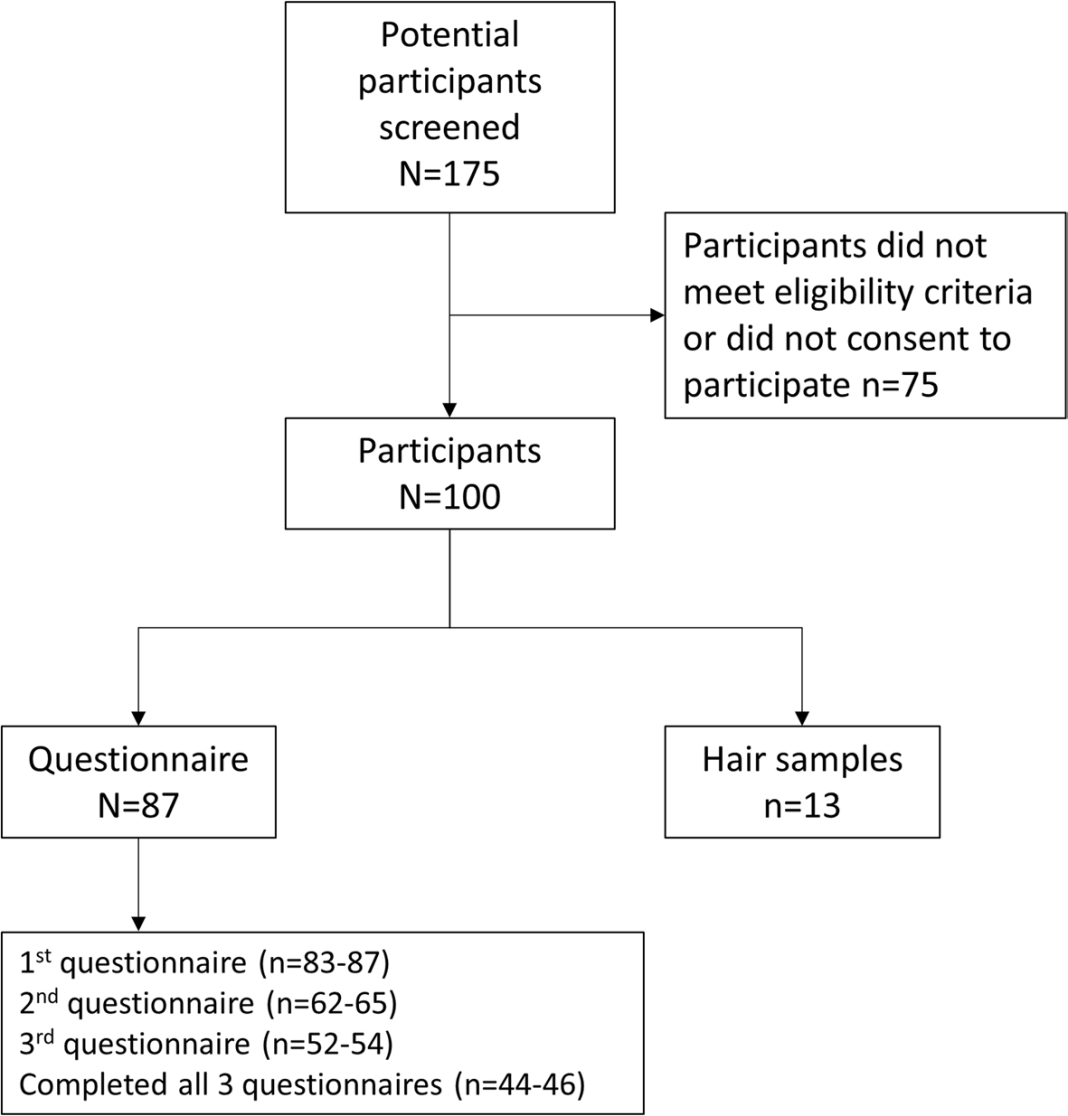
Flow chart indicating the number of women screened and the number of participants in different elements of the study. The total number of participants for individual components is greater than 112 because some women participated in more than one part of the study e.g. questionnaire and hair sample

Participation in the elements of the study is shown in Fig. 1. Eighty-seven women completed the questionnaire element of the study and a further 13 women gave hair samples, Of the 87 women who completed the initial questionnaire at 15 weeks’ gestation, 62-65 completed the questionnaires at 32 weeks’ gestation and 52-54 completed the questionnaires 6 weeks postnatal. The 100 participants were between 21 and 46 years of age at enrolment to the study (Table 1). The majority of women were of White British ethnicity; the sample included women from other White groups, Black African, South Asian and other ethnic groups. The largest group of women were married, although a large proportion described themselves as single. The majority of women were in paid employment, although a large proportion of women did not report this information. The reasons for not completing subsequent questionnaires included pregnancy loss, giving birth before 32 weeks’ gestation and loss to follow-up. As some scores were missing individual components between 44-46 participants completed all three questionnaires.

**Table 1:** Demographic characteristics of study participants (n=100)

Characteristic	Median (range) for continuous data N (%) for categorical data
Maternal age (years)	33 (21-46)
Body Mass Index (kg/m^2^)	26 (18-37)
Ethnicity	
* Bangladeshi*	1 (1)
* Black African*	3 (3)
* Far Eastern*	1 (1)
* Indian*	2 (2)
* Mixed*	1 (1)
* Pakistani*	3 (3)
* White British*	69 (69)
* Other White Ethnicity (Irish/European)*	9 (9)
* Other Ethnic Groups*	3 (3)
* Ethnicity not recorded*	8 (8)
Marital status	
* Single*	28 (28)
* Married*	44 (44)
* Other (Co-habiting / separated)*	9 (9)
* Status not recorded*	19 (19)
Employment status	
* Employed*	63 (63)
* Not in paid employment*	7 (7)
* Home maker*	6 (6)
* Not recorded*	24 (24)

**Fig. 2:**
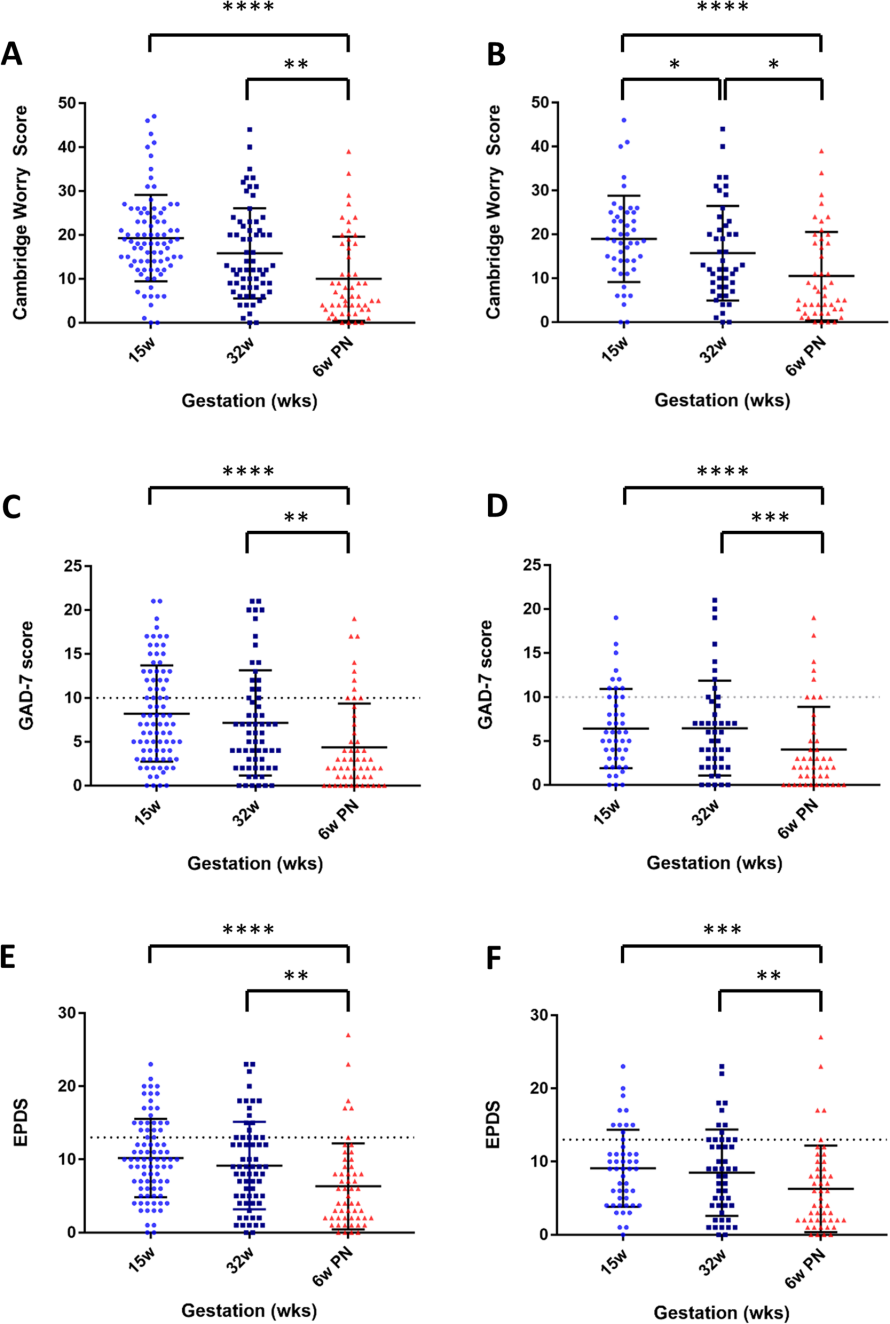
Results from psychometric questionnaires. **A**) Cambridge Worry Score (CWS) completed at 15 weeks’ gestation, 32 weeks’ gestation and 6 weeks postnatal for all participants, **B**) CWS completed at the same gestations for participants who completed questionnaires at all three time points, **C**) GAD-7 completed at 15 weeks’ gestation, 32 weeks’ gestation and 6 weeks postnatal for all participants, **D**) completed at the same gestations for participants who completed questionnaires at all three time points, **E**) EPDS completed at 15 weeks’ gestation, 32 weeks’ gestation and 6 weeks postnatal for all participants, **F**) EPDS completed at the same gestations for participants who completed questionnaires at all three time points. * p<0.05, ** p<0.01, *** p<0.005, ****p<0.001

Cambridge Worry Score (CWS) demonstrated a reduction from the highest levels at 15 weeks’ gestation to lower levels at 6 weeks postnatal (Fig. 2A), when analysis was restricted to participants who returned all three questionnaires (n=46) there was a stepwise reduction between the three time points studied (Fig. 2B). The profile of GAD-7 scores similarly found with the lowest levels seen at 6 weeks postnatal, but there was no difference in GAD-7 scores between 15 and 32 weeks’ gestation (Fig. 2C and D). Applying a threshold for significant anxiety of a GAD score ≥10 found 38% of participants had significant anxiety at 15 weeks’ gestation, 27% at 32 weeks’ gestation and 23% at 6 weeks postnatal. EPDS was again lowest at 6 weeks postnatal compared to during pregnancy, however, there was no reduction between scores at 15 and 32 weeks’ gestation (Fig. 2E and F). Applying a threshold value >13 in the EPDS found 30.1% had significant depressive symptoms at 15 weeks’ gestation which fell to 27.7% at 32 weeks’ gestation and 11.1% postnatally. The profile of GAD-7 and EPDS scores was the same when analysis was restricted to participants who returned all questionnaires (Fig. 2D and F). The strongest correlation was seen between GAD-7 and EPDS scores (r^2^ = 0.65), with weaker relationships between GAD-7 and CWS (r^2^ = 0.19) and EPDS and CWS (r^2^ = 0.17).

**Fig. 3:**
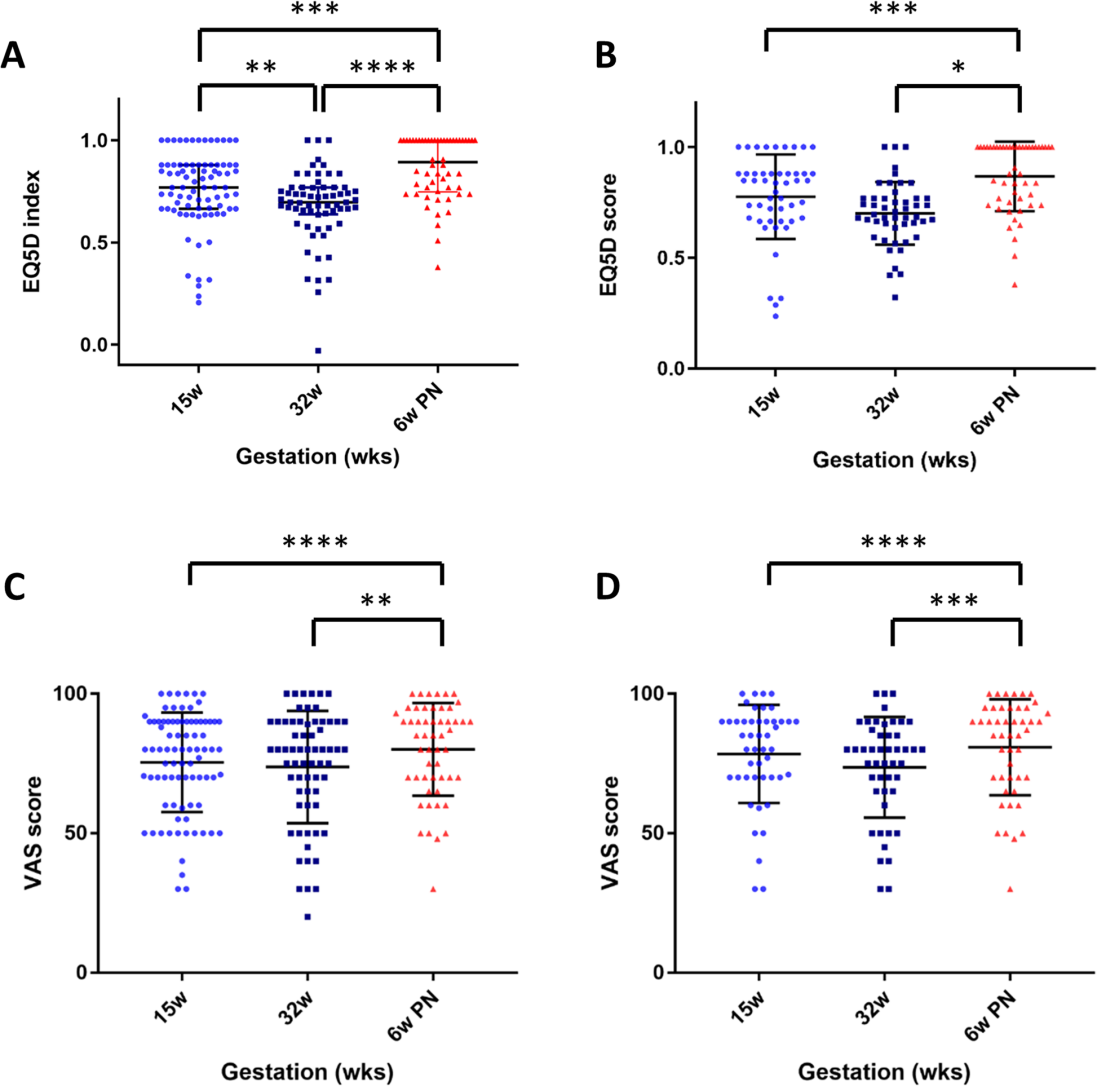
Results from quality of life questionnaires. **A**) EQ5D score completed at 15 weeks’ gestation, 32 weeks’ gestation and 6 weeks postnatal for all participants, **B**) EQ5D Score completed at the same gestations for participants who completed questionnaires at all three time points, **C**) Visual Analogue Score (VAS) for quality of life completed at 15 weeks’ gestation, 32 weeks’ gestation and 6 weeks postnatal for all participants, **D**) Visual Analogue Score (VAS) for quality of life completed at the same gestations for participants who completed questionnaires at all three time points. * p<0.05, ** p<0.01, *** p<0.005, ****p<0.001

**Fig. 4:**
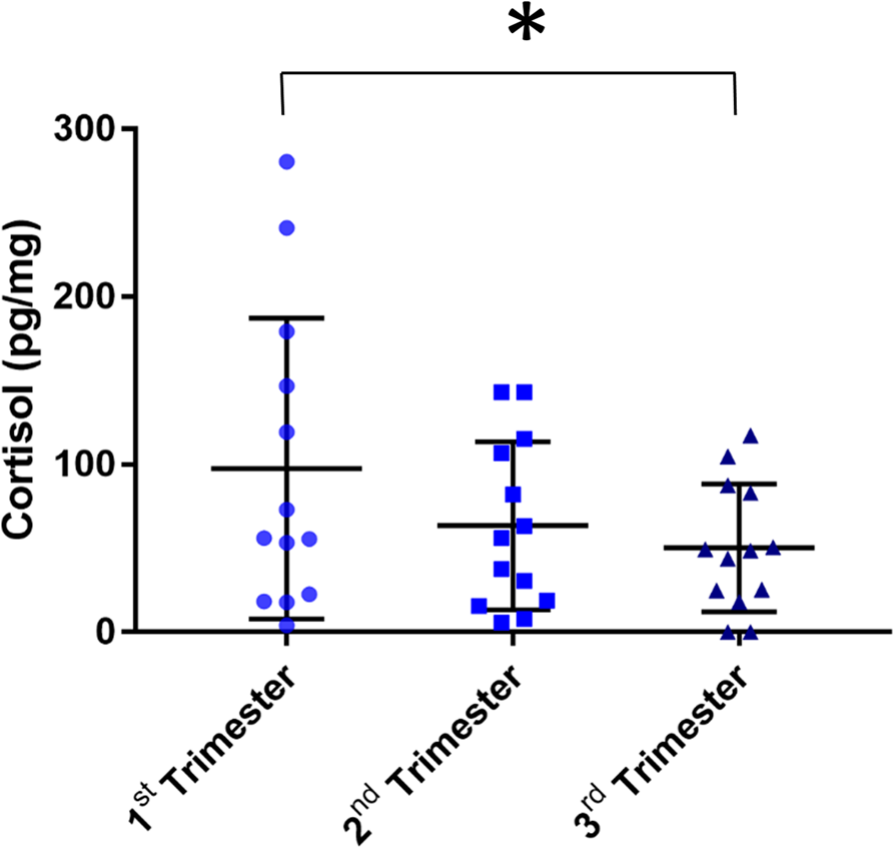
Hair cortisol measurement in pg/ml demonstrating a reduction in hair cortisol levels from the 1^st^ through to the third trimester. * p<0.05

The EQ5D-Index fell from 15 weeks’ gestation to a nadir at 32 weeks’ gestation with the highest levels of quality of life reported at 6 weeks postnatal (Fig. 3A and B). The responses to the visual analogue score about quality of life suggested higher scores at 6 weeks postnatal, which was statistically significant when analysis was restricted to women with complete questionnaire responses (Fig. 3C and D). There was a negative relationship between EPDS scores and quality of health as assessed by the visual analogue score (r^2^ = 0.12). Hair cortisol levels were reduced by 48% in the third trimester compared to the first trimester (Fig. 4).

## Discussion

This study demonstrates that measures of anxiety, depression, perception of quality of life and health status and stress in mothers alter at different time points in pregnancies after loss and in the early postnatal period. In general, levels of anxiety, depression and stress were at their highest levels in the first half of pregnancy and at their lowest in the postnatal period. Whereas, perception of health status and quality of life appeared to be at its lowest point in late pregnancy. Where thresholds were available over one-third of participants had significant symptoms of anxiety or depression.

### Strengths and Limitations

This study is strengthened by the combination of quantitative measures of maternal anxiety and depression which have been validated for use in pregnancy. However, this study did not employ a comparative design so conclusions cannot be drawn about the levels of anxiety or depression in comparison to pregnant women without a history of stillbirth. As not all women participated there may be a selection bias which could impact upon the responses. Similarly, not all women completed all three questionnaires, in some cases this was because women had given birth before 32 weeks’ gestation, and in other cases because a mother didn’t participate or moved from one of the participating units. In both cases, it is plausible that non-participants may have had higher levels of anxiety or depressive symptoms which would underestimate the impact of prior stillbirth on these symptoms in a subsequent pregnancy.

### Contextualising the findings

The importance of research into care in pregnancies after loss was emphasised by its inclusion into stillbirth research priorities identified by both parents and professionals [17]. Although there is a wealth of evidence that pregnancy after loss requires additional antenatal care in terms of support from professionals and investigations to identify recurrent or related conditions to help parents navigate the increased risk of psychological and medical complications [7], there are few studies that evaluate the impact of specialist antenatal services [11]. Warland and O’Leary identified that “support and early intervention at the time of stillbirth and subsequent pregnancy is likely to be useful. Further research is needed to determine whether early intervention can alter the tendency to paradoxical parenting style [10].”

The findings presented here provide a view of the psychological profile, levels of stress and perceived quality of life in women attending a specialist antenatal service for perinatal loss. Whilst this study was carried out in three sites in the North-West of the UK the findings are similar to those reported elsewhere, indicating that the findings are likely generalisable. Hughes and Turton reported average EPDS scores of 10.2 in the third trimester of pregnancy after stillbirth and 28% scored “high”, which was comparable to 9.2 and 28% in our sample respectively [18]. Our findings of increased symptoms of anxiety and depression are also in agreement with other earlier reviews describing symptoms in pregnancy after loss [19]. Interestingly, our data suggest that the levels of symptoms of anxiety and depression are highest at 15 weeks’ gestation and then fall as pregnancy progresses. This change could reflect increased belief that the pregnancy will result in a healthy outcome, or that contact with specialist antenatal service reduced the incidence of these symptoms; a study of the CWS in Spain found a significant reduction in the major worry that there was a possibility of something being wrong with the baby from 72.9% of respondents in the first trimester to 39.3% in the third trimester, whereas other issues such as going to hospital or coping with the new baby showed little change [20].

The levels of EQ5D-Index were comparable with postnatal measures from other UK studies. The 35-39 trial postnatal measure was 0.87 (standard error ±0.01) and was 0.89 (standard error ±0.02) in our study population [21]. However, the EQ5D index at 32 weeks’ gestation in women with a prior perinatal was lower than the point of randomisation of the 35-39 trial of healthy women over 35 years of age (0.70 vs. 0.84) which may indicate additional physical or mental health concerns in women with a history of stillbirth. Further evidence that symptoms of significant depression are linked to lower EQ5D-index scores is found in the WENDY study of 545 women. Pregnant women with significant depressive symptoms had a lower score than those without (0.90 vs. 0.70) [22], although the study also recognised that SF-12 may be a better reflection of quality of life. Therefore, addressing symptoms of depression and anxiety in this population may significantly improve health status.

During the study we also introduced measurement of hair cortisol as evidence emerged that this may reflect prenatal stress and relates to progression of depressive symptoms [23, 24]. The values of hair cortisol showed wide variation, but were in keeping with previously reported levels [23] and showed the same downward pattern as seen in the psychometric questionnaires. Interestingly, this is the opposite pattern to a study describing hair cortisol levels in uncomplicated pregnancies [25]. This method is evolving, and there is a need to standardise collection, storage and measurement techniques so that results can be compared between studies [23]. Nevertheless, this appears to be an objective means by which prenatal stress could be measured in women in pregnancy/ies after stillbirth but further work is required to determine whether the values obtained in this population differ from women without a history of loss.

Pregnancies after loss are associated with increased resource use [26], Hutti et al. described that resource use was particularly associated with increased maternal anxiety and depressive symptoms [27], such as those demonstrated in our population. One potential reason for the increased resource use is that women whose care needs are not being met, may have increased anxiety and depression and also seek additional appointments from healthcare professionals [7]. A qualitative study of 10 women attending a PALC in Brisbane, highlighted that mothers cited the importance of their relationship with clinical staff “who understood their previous loss and the associated anxiety” and were able to monitor for signs and symptoms of depression [28]. A priority setting exercise of 79 professionals involved in stillbirth research reported that targeted interventions for pregnancy after loss and specialist clinical services such as PALCs were identified as urgent and important by 79% and 73% of respondents respectively. Importantly, fewer 50% of respondents of respondents felt that randomised controlled studies were the best way to evaluate psychological interventions, or were likely to be feasible [29]. Nevertheless, models of care in pregnancy after loss need to be developed and evaluated in order that effective care can be delivered to women and their families in these pregnancies. In recent years, descriptive studies have provided a basis for improving bereavement care after perinatal death [2]; employing similar approach provides a means by which models of care for subsequent pregnancy can be developed and their impact on women and their families can be described.

## Conclusions

This study demonstrates heightened anxiety, stress and depressive symptoms which decrease as pregnancy progresses. Provision of specialist antenatal, intrapartum and postnatal care in a dedicated pregnancy after loss service is one potential means by which this these symptoms can be addressed. Studies are required to determine whether this model is superior to routine high-risk care and to identify which components of the dedicated service are valued. Further studies are also needed to understand partners’ and other family member’s experiences of pregnancy/ies after stillbirth to appreciate which aspects of care and support are beneficial in a future pregnancy.

## Data Availability

The datasets generated and/or analysed during the current study are not publicly available as ethical approval was not sought for their dissemination but are available from the corresponding author on reasonable request.

## Abbreviations

ANOVA: Analysis of Variance
CWS: Cambridge Worry Score
EQ5D: EuroQoL-5 dimension
EPDS: Edinburgh Postnatal Depression Score
GAD-7: Generalized Anxiety Disorder (7-item)
PALC: Pregnancy After Loss Clinic
UK: United Kingdom
VAS: Visual analogue scale
